# Reducing socio-ecological conflict using social influence modelling

**DOI:** 10.1038/s41598-022-26570-8

**Published:** 2022-12-20

**Authors:** Corrine M. Condie, Karen A. Alexander, Elizabeth A. Fulton, Joanna Vince, Scott A. Condie

**Affiliations:** 1 Institute for Marine and Antarctic Studies, University of Tasmania, Hobart, Australia; 2 Centre for Marine Socioecology, University of Tasmania, Hobart, TAS Australia; 3 International Centre for Island Technology, Heriot-Watt University, Stromness, Scotland; 4 CSIRO Environment, Hobart, TAS Australia; 5 School of Social Sciences, University of Tasmania, Launceston, TAS Australia

**Keywords:** Environmental social sciences, Ocean sciences, Mathematics and computing

## Abstract

Polarisation of opinions across communities can lead to social conflict, reputational damage and the disruption of operations and markets. Social influence models have been widely used to better understand processes driving conflict from a theoretical perspective. Using aquaculture as a case study, we demonstrate how such models can be extended to accurately hindcast the transition from population consensus to high conflict, including observed catastrophic tipping points. We then use the model to quantitatively evaluate strategies aimed at reducing aquaculture conflict. We found that persuasive advocacy was ineffective and often counterproductive, whereas meaningful engagement, collaborative learning and improving scientific literacy targeted broadly across the population was effective in moderating opinions and reducing conflict. When such messaging was targeted too narrowly or too infrequently, it tended to be negated by ongoing exchange of misinformation within the population. Both the modelling approach and lessons on effective communication strategies are relevant to a broad range of environmental conflicts.

## Introduction

Management of shared resources often requires stakeholders, with widely divergent value systems and interests, to work together cooperatively. Conflict occurs when these stakeholders have incompatible values or opinions^1,2^, competing ideas on the use of resources^3^, or when decision-making involves too few options^1^. Conflict may also arise where there is increased competition for ocean and coastal goods and services driven by changing socio-ecological conditions (e.g. population pressure, climate change, growth imperative), or where the political and social factors that shape the rules, rights, and effects of human resource use are inadequate^4^. Such conditions can result in a polarised discourse, whereby social groups adopt internally aligned views that diverge over time and are openly opposed to each other externally^5–8^. This behaviour is apparent in a range of environmental management and policy areas, including climate change^9^, renewable energy^10^, mining^11^, water resources^12^, forestry^5^, fisheries^13,14^ and aquaculture^15,16^, where it has been identified as one of seven global megatrends with potential for substantial and transformative impact^17^. The *Global Atlas of Environmental Justice* (www.ejatlas.org) currently documents more than 3700 instances of environmental conflict from the perspective of environmental advocates^18^. The impact of environmental conflict can affect all parts of society by disrupting decision making, impeding operations, imposing significant business costs, fragmenting communities and triggering violence^5,8,19,20^.

Over the past decade, aquaculture has emerged as a highly contested issue worldwide. As fishery catches have exceeded sustainable levels in many regions, aquaculture has expanded rapidly with global production now surpassing wild-catches^13,21^. This growth has generated a high level of community concern relating to issues such as the utilisation of coastal and marine space^22^, contamination of aquatic environments^23^, interactions with native species^24^, and fish welfare^25^. The growth of salmon aquaculture has been particularly contentious, with more than two thirds of global salmon production now farmed. In countries such as Australia^15,26^, Canada^27^, Chile^28^, Norway^29^ and Scotland^30^, opposing stakeholder views on the growth and development of salmon aquaculture has resulted in the consolidation of pro-industry and precautionary groups, characterised by entrenched positions impervious to rebuttals or alternative viewpoints. This conflict is limiting industry growth and challenging many of the prevailing governance arrangements for the sector. In its most extreme form, debate in Chile has escalated into social unrest, violence and destruction of property^28,31^.

While the growth of conflict around salmon aquaculture has been well documented, there has been little progress in understanding the underlying drivers and social dynamics of the debate. This has hindered quantitative analyses and precluded development of any forecasting capability or rigorous evaluation of potential interventions. This poses two fundamental research questions. How can the underlying social dynamics of such a system be modelled and what can such a model reveal about the efficacy of communication strategies aimed at reducing conflict levels. Here we have used the Australian salmon industry as a case study to track the evolution of conflict^15,16,32^ and explore the role that communication can play in mediating debate^33–35^. This industry is located wholly within the island state of Tasmania, with debate evolving within a well-defined community largely isolated from external influences. A relatively stable political environment also reduces the risk that the debate may have been confounded by interests or grievances extraneous to industry issues.

**Figure 1:**
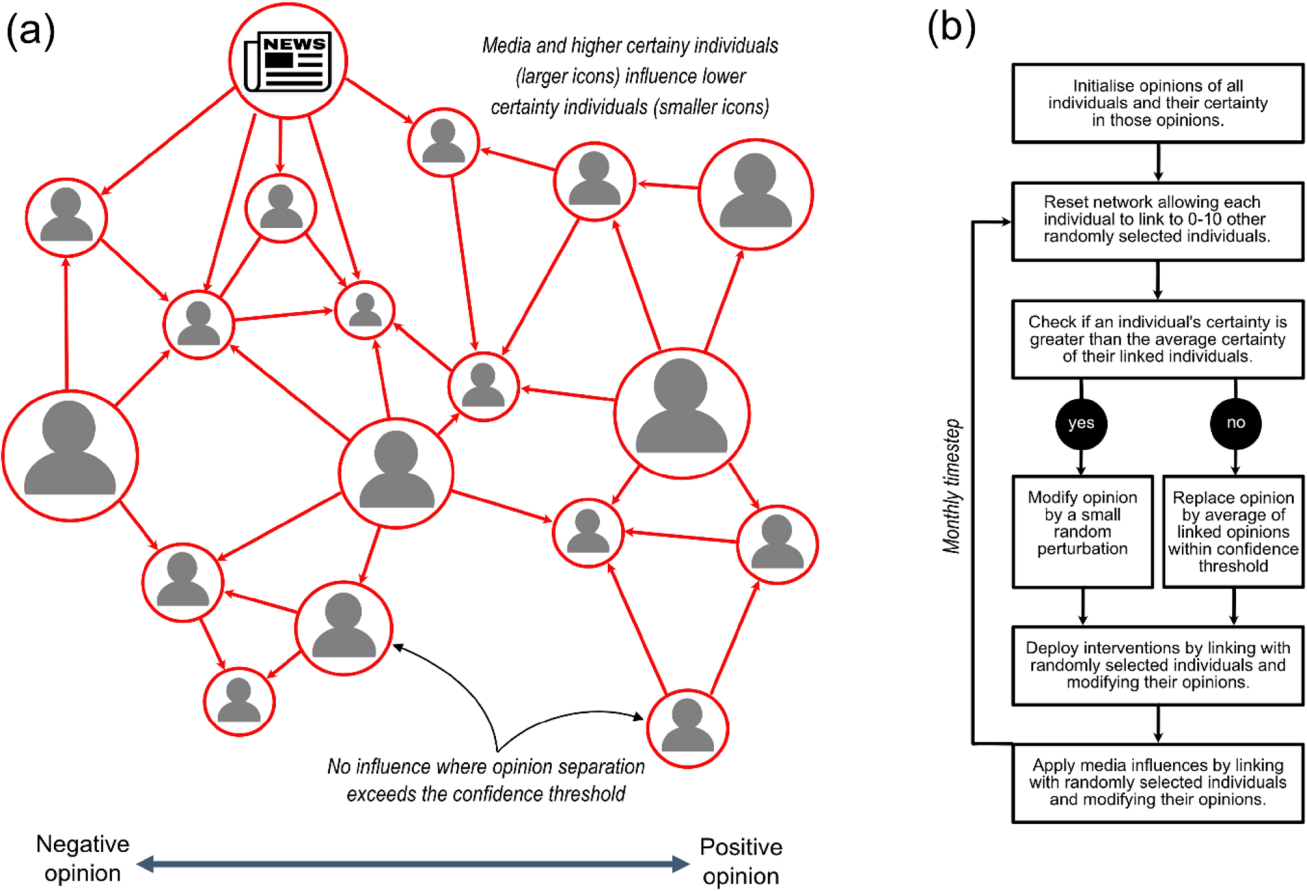
(**a**) Basic network structure of the Social Influence and Events Model (SIEM). The opinion of an individual can potentially be influenced by media or other individuals with higher average certainty (larger icons) and opinions that fall within their confidence threshold (separation of icons). Because homophily is a system characteristic, confidence threshold is the same for all individuals, whereas opinion and certainty vary between individuals. (**b**) Processes simulated in SIEM on a monthly timestep.

An ensemble agent-based social influence model (Fig. 1) has been used to successfully simulate the development of social conflict from the Tasmanian salmon industry’s inception in the early 1990s, to present day. The model also provided a robust framework with which we were able to explore the transition from consensus to conflict, including delineation of system tipping points. Finally, it was used to test the extent to which alternative communication strategies were able to reduce conflict levels–a function which is particularly pertinent in high conflict situations where poor strategy choice can result in increased volatility and unintended consequences.

## Methods

### Study region and industry development

The Australian salmon industry is located in Tasmania, an island state of 550,000 inhabitants. Tasmania has a well-documented history of environmental campaigning centred around issues such as forestry^36^, hydro-power generation^37^, trawler fishing^38^, and salmon farming^16^. It is also the birthplace of a national environmental movement and political party^39^, which have successfully lobbied to introduce major reforms to environmental policy over the past three decades^40^. Salmon farming commenced in Tasmania in 1986 and has expanded rapidly over the last decade to become the state’s largest primary industry, with the current value of US$0.7bn forecast to double by 2030^41^. This rapid expansion has been accompanied by increasing conflict, with many in the community questioning the ecological impact and governance of the industry^15,16,32^. The three main areas of contention are: inadequate information and communication processes; the perceived lack of science-based decision-making; and the failure of managing agencies to enforce existing regulations on pollution and environmental sustainability^15,42^. Post 2016 there has been a significant increase in anti-salmon farming sentiment, supported by well-organised protest actions^15,16^ and launch of the book *Toxic: the rotting underbelly of the Tasmanian salmon industry*, written by an internationally renowned Tasmanian author, Richard Flanagan^43^. Importantly for this research, the evolution of this conflict has been well documented with: (i) two government inquiries into the Tasmanian salmon industry (used here to identify conflict issues^15^); (ii) an active broadcast media, reporting on events over the length of the industry life cycle (used here to force the model^26^); and (iii) 5 state-wide community opinion surveys (used here to validate the model).

### Model framework

The evolution of views on the Tasmanian salmon aquaculture industry were modelled using the Social Influence and Event Model (SIEM, Fig. 1)^7^. This agent-based network model is an extension of the seminal Hegselmann-Krause (HK) bounded confidence model^44^ that tracks the opinions of individuals through time as they are influenced by other individuals in the community. In addition, SIEM incorporates the broader influence of mass media across the community (Tables S1 and S2). While social influence models have previously been used to explore a diverse range of social interactions, this study introduced two important advances. The first was to demonstrate accurate and robust hindcasting of surveyed community opinions at the population level. The second was to quantitatively evaluate alternative communication strategies aimed at moderating opinions and reducing conflict.

### Influence between individuals

After testing computational feasibility and confirming previous findings^7^ that model results were insensitive to network characteristics, such as population size (Table S3, Fig. S2) and number of influencing links (Table S3, Fig. S3), a representative population of 350 individuals was adopted for most model runs (Table 1). The opinions of individuals were assumed to be continuous (rather than being limited to discrete choices^45^) and at each timestep could be influenced by the opinions of other individuals in the network (Fig. 1a, Tables S1 and S2). Linkages between individuals changed randomly at each timestep and were limited to pairs of individuals whose opinions were not too far apart (Table S2, Eq. S2). This constraint is known as *homophily* and the maximum separation of opinions allowing individuals to influence each other is referred to as the *confidence threshold*. The confidence threshold was found to be the only parameter to which model results were sensitive (Table S3, Fig. S4) and its value was therefore determined through a heuristic model calibration process.

**Table 1:** Model parameters and strategies for capturing parameter uncertainty. Where a range is indicated, values were assigned randomly at each time-step during the model run. For example, newspaper articles were assumed to reach 0–40% of the population. The insensitivity of model results to network characteristics, such as population size and number of linkages, allowed smaller populations to be utilised in the model.

Variable	Values	Model sensitivity	
Population size	350	SIEM previously found to be insensitive to this parameter^7^, which has been confirmed for the current application (Figure S2)	
Links per month between community members	[0 0.03] of population	SIEM previously found to be insensitive to this parameter^7^, which has been confirmed for the current application (Figure S3)	
Links of community members with newspaper articles (or the events they relate to)	[0 0.40] of population	Results previously found to be insensitive to this parameter^7^ and values were relatively well constrained by newspaper readership within Tasmania	
Certainty probability distribution for population	Triangular distribution with range [0 1] and mode 0.3	Analysis of a diverse set of distributions (Table S3) indicated that triangular distributions replicate historical trends satisfactorily and are relatively insensitive to mode. Flat and binary distributions produced larger errors	
Confidence threshold (level of homophily in the community)	0.4 of the opinion range	Model results were sensitive to the confidence threshold (Figure S4). Values close to 0.4 produced realistic opinion trajectories. However, values < 0.35 produced opinions that were more tightly coupled and volatile, responding rapidly to newspaper coverage with less opportunity to develop conflict. Values > 0.45 produced opinions that were more insulated and stable, responding only weakly to newspaper coverage with low conflict levels maintained indefinitely	
Framing (opinion) of newspaper articles derived using QCA	Very negative Negative Neutral Positive Very positive	[-1.0 -0.5] [-0.5 0.0] 0.0 [0.0 0.5] [0.5 1.0]	Values were varied randomly within the specified ranges with associated model sensitivity represented by the range of individual ensemble results (Fig. 2b,c)
Influencing strength (certainty) of a newspaper article derived using QCA	Very negative Negative Neutral Positive Very positive	[0.5 1.0] [0.0 0.5] 0.0 [0.0 0.5] [0.5 1.0]	Values were varied randomly within the specified ranges with associated model sensitivity represented by the range of individual ensemble results (Fig. 2b,c)
Influencing strength (certainty) of communication strategies	Influential (0.5) Highly influential (0.8)	Model sensitivity represented by differences in intervention scenario results (Fig. 3a)	
Frequency of communication interventions	Monthly, quarterly	Model sensitivity represented by differences in scenario results (Fig. 3b)	

At each timestep (Fig. 1b), individuals changed their opinion only if their *certainty* in that opinion (details below) was less than the average certainty of other individuals with which they were linked^46^ (Table S2, Eq. S4). When this condition was satisfied, the opinion of an individual was replaced by the average of their own opinion and the opinions of all other influencing individuals (i.e. *assimilative influence*: Table S2, Eq. S1). In addition, small random fluctuations in the opinions of individuals (0–10% of the confidence threshold) allowed opinions to drift and occasionally move inside the confidence threshold of other individuals. In the absence of other influences (such as mass media), these random fluctuations always result in sufficient connections to eventually overcome homophily and drive convergence of opinion clusters that may have initially formed due to homophily^47^.

### Certainty of individuals

Imposing a distribution of certainty levels across the community recognised differences not only in individual’s experiences, knowledge and access to relevant information^46^, but also personal characteristics such as persuasiveness, social status, open-mindedness and self-belief^48^. Ranking of certainty was also consistent with experimental findings indicating that influential individuals also tend to be less susceptible to the influence of others^49^.

For most model runs, certainty was distributed across the population according to a simple triangular probability distribution skewed towards lower certainty (range [0 1] and mode 0.3). This distribution is consistent with results from a recent community survey conducted by the authors. In any case, results were largely insensitive to the detailed structure of the distribution (Table S3, Fig. S1).

At each time-step, the certainty of individuals drifted randomly: first by taking a random value between their current certainty and their initial certainty; and second by shifting randomly in either direction by less than 5% of the allowable opinion range^7^. Constraining the range of each individual’s certainty in this way reflected its aforementioned dependence on relatively stable personal characteristics.

### Influence of mass media

Previous exploration of the model behaviour considered the influence of random events^7,50^, whereas here we model the influence of newspaper media as a proxy for events relating to salmon aquaculture in Tasmania. Newspapers are widely read by the Tasmanian population and known to be highly influential across the broad community^51,52^. Because there were no other events represented in the model, we effectively assumed that all relevant influential events were captured by Tasmanian newspaper articles. However, this does not preclude influence by newspaper articles that were not directly connected to an external event, such as editorials or other opinion pieces that commonly relate to aquaculture.

All articles from Tasmanian’s three daily newspapers appearing over the past 25 years (March 1996 to March 2021) have previously been analysed using *qualitative content analysis* (QCA) and their *framing* categorised on a 5-point Likert scale (very negative, negative, neutral, positive, very positive^26^). Use of the QCA process within this context was validated using the *content analysis trustworthiness checklist*, whereby the categories and coding rules were externally audited and coders were trained to ensure consistency in ratings^53^. The framing was then used to allocate an effective ‘opinion’ and an effective ‘certainty’ to each article (Table 1).

This formulation allowed model events to influence individuals in the same way that individuals influenced each other, with events differing only in their ability to simultaneously influence a large proportion of the population within a single timestep. For example, an article with a very negative framing could potentially move opinions of readers towards a more negative opinion provided their existing opinion was within the range imposed by homophily and their certainty was less than the certainty associated with the article.

### Testing communication strategies

The model was used to test the efficacy of a range of communication strategies in terms of their ability to change opinions and reduce conflict. Each strategy was characterised by an opinion, a certainty, a frequency (times deployed per month) and in some cases a target subpopulation defined in terms of certainty levels (Table 2). Communication strategies were assumed to influence individuals in the same way as media articles, although unlike media, strategies were applied at a specified frequency throughout the deployment period and could be targeted.

**Table 2:** Definition of communication strategies tested within SIEM. Parameters not defined here were as in Table 1, with messaging assumed to reach 0–40% of the population (i.e. same range as newspaper articles).

Communication strategy	Message opinion	Message certainty	Message frequency	Message targeting
Meaningful engagement & collaborative learning	Population average for that month	0.5	4 per month	Entire population
Enhanced marine literacy	Neutral (0)	0.8	4 per month	Entire population
Negative persuasion	Strongly negative (−0.8)	0.5	4 per month	Entire population
Positive persuasion	Strongly positive (0.8)	0.5	4 per month	Entire population
Positive & negative persuasion	Strongly positive & strongly negative (± 0.8)	0.5	4 per month	Entire population
Less frequent (positive persuasion)	Strongly positive (0.8)	0.5	4 in the first month of each quarter only	Entire population
More targeted (enhanced marine literacy)	Neutral (0)	0.8	4 per month	Individuals in upper 15^th^ percentile of certainty

Three aspects critical to any communication strategy were tested (Table 2): (a) how the message should be framed—positive persuasion supporting industry, negative persuasion supporting community concerns, neutral framing aimed at enhancing scientific literacy, or meaningful engagement and collaborative learning effectively supporting the current mean opinion of the population; (b) how frequently the message should be deployed—monthly or quarterly campaigns; and (c) how the message should be targeted—broadly across the population or focused on key influencers.

### Simulations

The model used a monthly time-step. Each month a new random network was generated with each individual forming links with 0–10 other individuals (5 on average, although results were largely insensitive to the number of links: Table S3, Figure S3). Opinions of all individuals were then updated according to the influences of both other individuals and newspaper articles relating to salmon aquaculture that appeared that month (as described previously).

All model runs started in January 1996 with the entire population holding near neutral opinions [−0.1 0.1]. Articles relating to Tasmania’s salmon aquaculture, first appeared in newspapers in March 1996, and the model was forced by all subsequent articles up until March 2021. From April 2021 to March 2032, projections were generated by repeating a 3-year cycle of newspaper articles from the period April 2018 to March 2021. Utilising this period assumed that issues related to salmon aquaculture will continue to be contested over the next decade. Stochastic variability in model responses was captured by running each scenario 500-times to form an ensemble that could be described statistically.

The opinions of all model individuals were tracked through time and conflict was defined as the standard deviation of opinions in the population at that time^7^ (Table S1). Results for both mean opinion and conflict levels were evaluated using reported qualitative evidence (prior to 2016) and independent survey results (2016–2021). The independent surveys were Tasmania-wide (population 550,000) and included an industry sponsored survey in October 2016 (1000 respondents)^54^, a newspaper poll in August 2017 (1000 respondents)^55^, a research survey in August 2020 (406 respondents)^56^, a second industry sponsored survey in June 2021 (1000 respondents)^57^, and a second research survey conducted by the authors in October 2021 (448 respondents).

Communication strategies were compared over the projection period (April 2021 to March 2032) in terms of their ability to both change opinions and reduce conflict. A total of seven scenarios are reported with differences in message framing, message frequency and message targeting (Table 2). While this set represents only a small fraction of potential scenarios, they cover the broad communication strategy options available and illustrate the general influence of message frequency and targeting observed in preliminary testing of the model using much smaller ensembles.

## Results

The results are presented in three sections: (i) simulating the evolution of opinions and conflict over the Tasmanian salmon aquaculture industry lifecycle; (ii) evaluating the robustness of the simulations by analysing model parameter sensitivities; and (iii) testing the efficacy of alternative communication strategies in reducing conflict levels.

### Simulating the evolution of opinions and conflict

**Figure 2:**
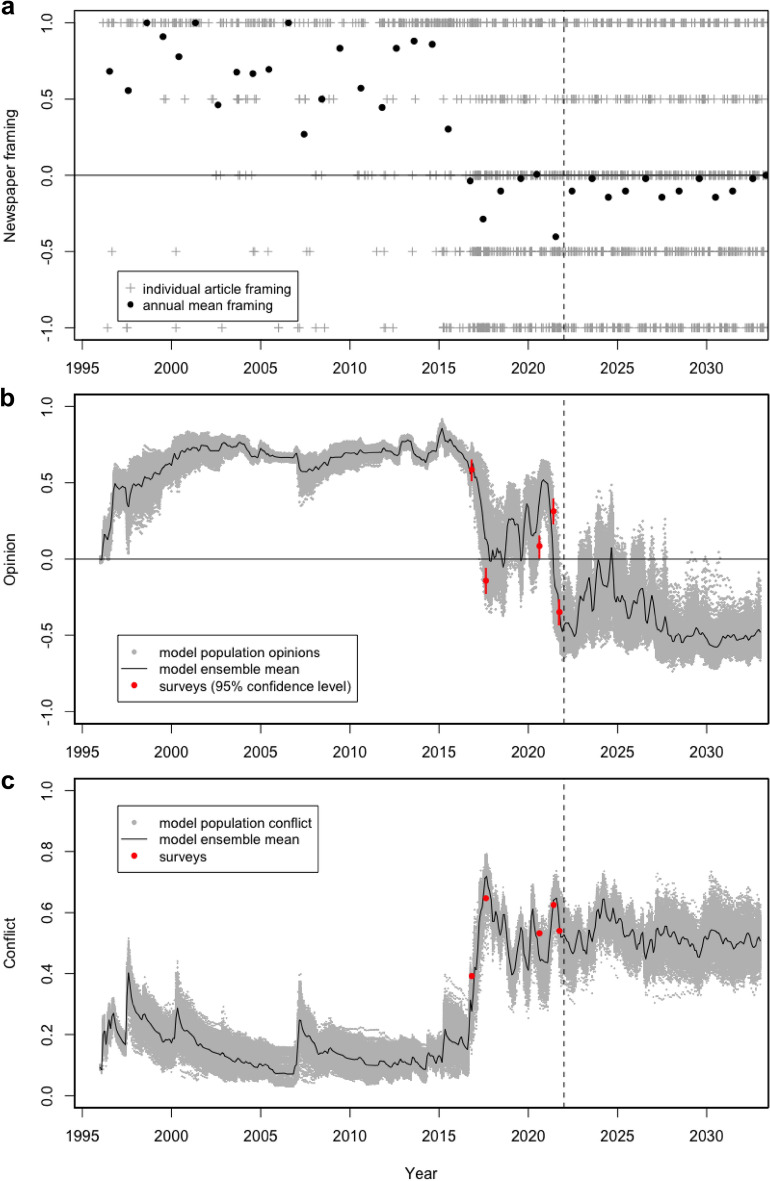
Time series over the historical period (January 1996 to March 2021) and a projection period based on repetition of the previous 3-years (April 2021 to March 2032). (**a**) Newspaper framing per article (grey crosses) and annual means (black points). (**b**) Modelled opinions averaged across the population (grey points) and averaged across the entire ensemble (black line). Average opinions estimated from five community surveys with 95% confidence intervals are also shown for comparison (red points). (**c**) Modelled population conflict levels (grey points) and ensemble mean conflict level (black line). Average conflict estimated from the five community surveys are also shown for comparison (red points). While no survey data was available prior to 2015, the industry has been described as having ‘an enviable social licence to operate’ ^58^ over that period, consistent with positive opinions and low conflict.

The evolution of newspaper coverage^26^, population opinion and conflict level over the industry lifecycle for Tasmanian salmon are shown for both a hindcast period (1996–2021) and a future scenario period that assumes continuation of recent media framing (2021–2032) (Fig. 2). While there was no quantitative data on community opinions available prior to 2016, the positive opinions (Fig. 2b) and low conflict levels (Fig. 2c) estimated by the model for that period are consistent with qualitative assessments describing the industry as having ‘an enviable social licence to operate^58^’. For the period 2016–2021 there is good agreement between model results and five independent Tasmanian state-wide surveys for both mean opinion and mean conflict^54–57,59^. Comparing survey results with the ensemble mean opinion yields a moderate level of agreement (NRMS error = 0.133; Spearman’s *r* = 0.7, *N* = 5, *p* = 0.233; Pearson’s *r* = 0.707, *N* = 5, *p* = 0.182; Table S3), while ensemble mean conflict yields stronger agreement (NRMS error = 0.073; Spearman’s *r* = 1.0, *N* = 5, *p* = 0.0167; Pearson’s *r* = 0.975, *N* = 5, *p* = 0.0047; Table S3). Correlations were ultimately limited by the small number of available surveys and volatility in both opinion and conflict over that period (Fig. 2b,c). However, all survey results fell within the ensemble range and major tipping points in population opinions and conflict in 2016 and 2021 were well resolved by the model.

Prior to 2015, opinions were stable and positive (Fig. 2b) with low levels of conflict (Fig. 2c). This pattern is consistent with the predominantly positive newspaper coverage (Fig. 2a). A tipping point in 2016 was pre-empted by a large shift in newspaper coverage towards a high proportion of negatively framed articles (Fig. 2a). The percentage of negative articles jumped from 8% prior to March 2015 to 43% between March 2015 and March 2021^26^. While the modelled community opinions were clearly influenced by the framing of Tasmanian newspaper articles, opinion dynamics within the community also had a large effect. For example, mean opinion remained strongly positive for almost 2-years following the media shift (Fig. 2b) due to underlying systemic resilience. Whereas conflict levels showed a more immediate response, with a modest increase in magnitude accompanied by much higher variability (Fig. 2c and individual ensemble points in Fig. 2b).

While newspaper framing became more negative from 2015 (Fig. 2a), the influence of those parts of the community that had remained positive became dominant whenever newspaper coverage waned. This resulted in moderately positive average opinion throughout much of 2020 and 2021 (Fig. 2b), albeit accompanied by high levels of conflict (Fig. 2c). Continuing predominantly negatively framed newspaper coverage over the projection period eventually moved the average opinion to moderately negative as individuals with lower certainty changed their opinion (Fig. 2b). However, positive opinions were sustained by a significant proportion of the population, so that conflict remained high (Fig. 2c).

### Model sensitivity

When population characteristics, such as the distribution of certainty across the population (Fig. S1), population size (Fig. S2) and population connectivity (Figure S3) were varied, model responses were robust with retention of key features including low conflict prior to 2015, rapid transition to high conflict in 2016, partial recovery through 2020, and finally further deterioration in 2021. However, model results were more sensitive to the confidence threshold (Figure S4). When the confidence threshold was set too low (< 0.35), opinions of individuals tended to be strongly aligned across the population and closely followed newspaper coverage. Resulting opinions were volatile, but there was little scope to develop conflict under these conditions. When the confidence threshold was set too high (> 0.45), individual opinions tended to be insulated from other influences and largely unresponsive to newspaper coverage. Population opinions then remained positive and conflict was again underestimated.

Calibration of the model was heuristic. As the only parameter exhibiting high model sensitivity, the confidence threshold was first tuned to yield satisfactory alignment with survey data (${\varvec{\varepsilon}}$ = 0.4). While results were far less sensitive to other parameters, the best agreement achieved with survey data used a population distribution skewed towards lower certainties (mode = 0.3, Table S1, Fig. 2), consistent with the notion that the majority of the population generally have limited insight into specific issues.

### Performance of communication strategies in reducing conflict

**Figure 3:**
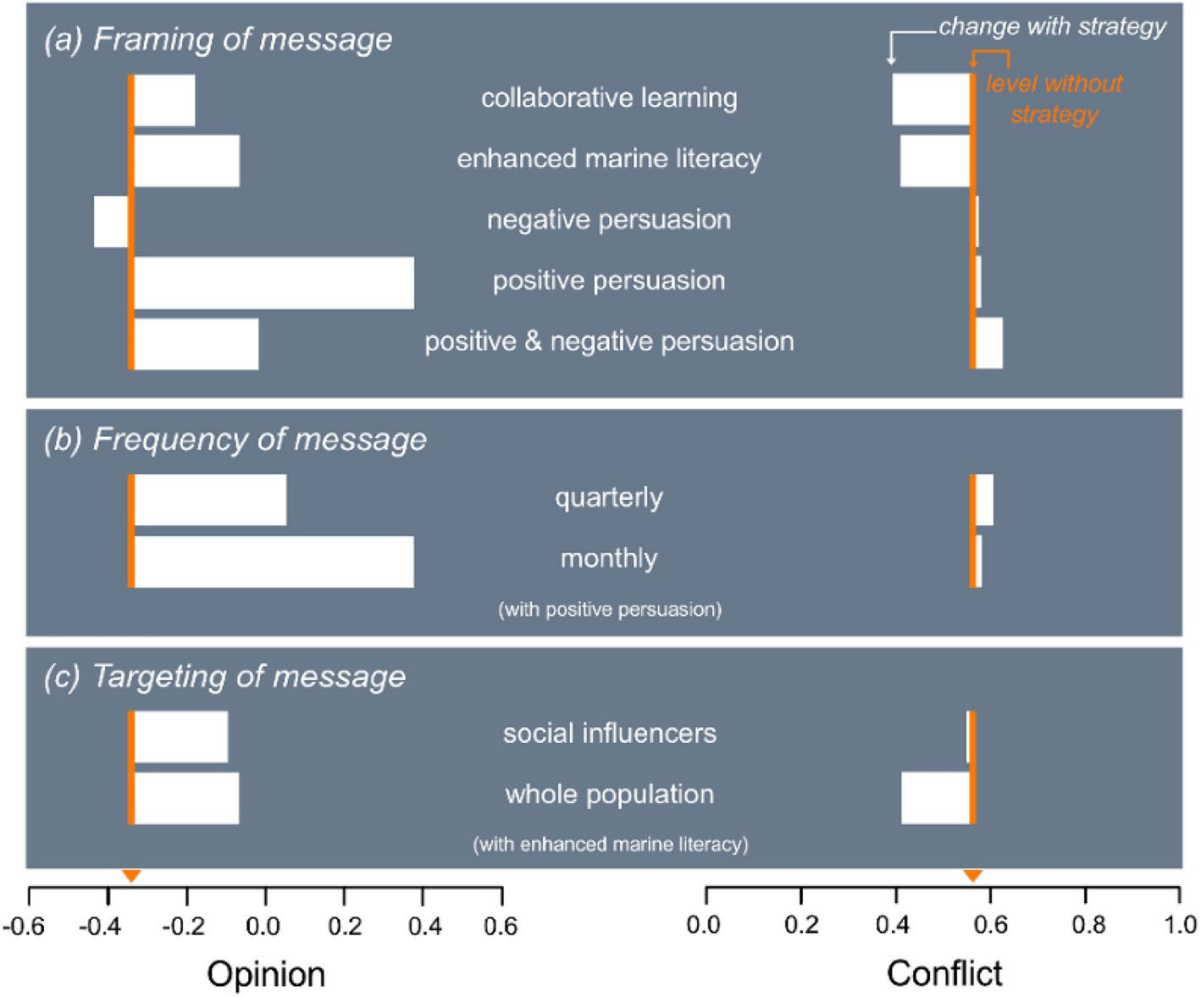
Movement of average opinion and conflict in response to a range of communication strategies applied from 2021. The orange line indicates baseline values for opinion and conflict when no strategy was applied (case shown in Fig. 2). Examples of time-series plots for collaborative learning and positive persuasion are shown in Fig. S5.

Comparing opinions and conflict levels (averaged over both the future projection period and ensemble runs, Fig. 3a, Fig. S5) reveals that framing of the message had a large influence on the efficacy of communication strategies. As expected, positive persuasion (e.g. low level advertising and advertorials) tended to shift opinions towards the positive and vice versa. The change due to positive persuasion was larger than for negative persuasion only because opinions started quite negative (Fig. 3a). When the frequency of positive persuasion was reduced from monthly to quarterly, its influence on average opinion also diminished, whereas less consistent messaging over time resulted in a small increase in conflict (Fig. 3b). Importantly, neither positive or negative forms of persuasion were effective in moderating conflict and when they were applied simultaneously conflict increased (Fig. 3a).

In contrast to persuasive approaches, both collaborative learning and enhancing marine literacy resulted in a decline in conflict, with average opinion moving towards a more neutral stance (Fig. 3a). However, the ongoing negative newspaper coverage continued to drive relatively large fluctuations in both opinion and conflict levels (Fig. S5).

When efforts to enhance marine literacy were focused only on social influencers (i.e. individuals with high certainty), the effect on the average opinion of the population was similar to that achieved when efforts targeted the entire population (Fig. 3c). However, this strategy was ineffective in reducing conflict (Fig. 3c), with ‘trickle-down’ dissemination to the broader population being too slow to significantly counter ongoing negative influences from newspaper coverage.

## Discussion

Polarised opinions and socio-ecological conflict are increasingly common across sectors such as mining^20^, energy^10^, forestry^5^, water management^12^, fisheries^13,14^ and aquaculture^16,26,60^. These conflicts have the potential to impose significant reputational damage, as well as triggering community action and regulatory responses that are disruptive to normal operations and markets^8,20^. There are various hypotheses in the literature as to what may have contributed to this escalation. For example: that the public’s lack of knowledge and understanding of industry practices has left them susceptible to misconceptions and disinformation promulgated by public interest groups^26,61^; that social media sites such as Facebook, Twitter, Blogspot and WordPress have blurred the boundary as to who should be regarded as an authority and what information constitutes evidence^16,62^; and that marked changes in agenda setting in broadcast media have led to reader ambiguity and uncertainty^16,26^. Although all are plausible, what is evident is that the recent proliferation of misinformation can confuse individuals and degrade societal trust^17^. In this climate, building community consensus will require a dialogue that is both constructive and informed, accurately representing and communicating real socio-ecological risks and addressing misconceptions^17,60^.

Social influence models have been widely used to better understand processes driving conflict from a theoretical perspective ^7,44,45,47^. Our study further demonstrates that they can provide governments, industry and researchers with the capability to hindcast and better understand the development of societal conflict. In the case of Tasmanian salmon aquaculture, the model has captured the development of conflict over the industry lifecycle, including the critical tipping points (as measured by the five community-wide surveys). SIEM indicates that while mean community opinion was resilient for almost 2-years following the change in media coverage (Fig. 2a,b), conflict showed a more immediate response (Fig. 2c). This response was variable across the model ensemble, but generally the recovery to lower conflict over 2015 was much slower than for a previous perturbation in 2007 triggered by the negative media associated with industry fines for polluting and a major fish escape (Fig. 2c). The 2015 transition appears to exhibit ‘critical slowing down’, whereby systems approaching a critical point recover more slowly from perturbations^63,64^. Importantly, when the 2017–2021 media forcing was continuously repeated over the future projection period, opinions never returned to 2017–2021 levels, but rather switched to a more negative stable state with persistently high conflict. This behaviour suggests that the tipping point may have been a so-called ‘catastrophic bifurcation point’, whereby the original state cannot be recovered except through another catastrophic transition^64^. This finding is particularly pertinent to the future management of the industry, as it suggests that to regain their ‘enviable social licence to operate’ the salmon industry may need to transition to a new industry lifecycle by moving farming operations either onshore or much further offshore.

The robust response of the model (Table S3, Figures S1, S2, S3) and good fit to historical survey data (Fig. 2) supports its use as a platform for pre-testing conflict reduction strategies. This is a particularly valuable capability in high conflict situations where trialling approaches within the community could have unintended and undesirable consequences. For example, there is extensive research indicating that low level advertising can change opinions, often more effectively than social media^65,66^. However, SIEM illustrates that it can be counterproductive in reducing conflict levels, particularly where it triggers persuasive counter-claims (Fig. 3a). Here, alternative strategies, directed at meaningful engagement, collaborative learning and enhancing scientific literacy, appear to be more effective.

Obtaining and maintaining societal trust will be a challenging task for many industries over the coming decade^17^. SIEM results support use of neutral highly credible spokespersons (Fig. 3a) to promulgate information to a broad cross-section of the community (Fig. 3c) with sufficient frequency to overcome any ongoing exchange of misinformation (Fig. 3b). For example, sustained science-based programs can promote science literacy, facilitate collaborative learning, and build consensus across stakeholder groups in relation to coastal ecosystem health^9,67^. If broadly supported, scientists can take an even broader role by interpreting information, leading informed debate, and identifying causality and responsibility where appropriate^15,67^. Boundary organisations can also play a key role in delivering such programs, as demonstrated in the Tasmanian context by the success of the Derwent Estuary Program^68^ and similar programs in other contested sectors such as forestry^5^.

Research indicates that public risk perceptions will be heightened where there is an information gap coupled with an active broadcast and social media emphasising adverse impacts. This situation is apparent in the Tasmanian salmon industry where a lack of meaningful engagement on issues of community concern left an information vacuum that was quickly filled by an active broadcast and social media emphasising negative environmental impacts^15,15^. This heightened perceptions of public risk until the tipping point was reached in 2016 (Fig. 2b). Recent industry forums have suggested that a way forward is ‘radical transparency’, where industry is seen to openly engage with its’ publics, using social media to ‘articulate their sustainability story^66^’. That is, to rebuild community trust both corporations and governing agencies should implement actions and approaches that radically increase the openness of both organisational process and data^67,69^. However, the real challenge is not one of transparency, but how to identify, synthesise and target relevant information to particular stakeholder groups such that it is seen to maintain its authenticity and credibility^70^. This will require both an understanding of stakeholder knowledge needs and communication networks, and the development of new innovative forms of communication and information streams^60^. There is also the need for both a common language between stakeholder groups, and an agreed set of sustainability indicators^66^.

SIEM demonstrates that relatively simple socio-ecological opinion models can accurately and robustly represent the evolution of population opinions in the real world. This effectively promotes such models from the realm of helpful theoretical constructs^7,44,47,71^ to applied strategic tools with a wide range of potential applications. The key element in achieving this step was recognition of the coupled role of social interactions and external mediatised events^7,26,50,72^. The approach can now be tested more broadly across other conflict issues, sectors and communities. While every application will have its own characteristics, the model structure is remarkably generic (Table S2) and in most situations should provide a practical starting point.

The main challenge to broader deployment will be accessing datasets needed to parameterise the model for real-world applications. For Tasmanian aquaculture, state-wide newspaper coverage appears to have adequately captured the influence of mass media. However, the media landscape around other conflicts may be more complex, requiring integration of data from diverse sources. Obtaining longitudinal data for model calibration may be even more challenging. Each of our estimates of opinion and conflict were based on responses from surveys of 400–1000 individuals sampled from the state’s population of almost 550,000. While it was fortuitous to have access to five such surveys over the period of evolving conflict, this still provided little scope to withhold data for independent validation of the model. Limited temporal coverage can also increase the challenge of fitting models to data and while we were able to achieve significance correlations for the Tasmanian salmon debate based on only five survey points (Table S3), other conflicts may involve a broader range of confounding factors. For example, the salmon conflict in Chile has been part of a much broader political debate influenced by a diverse range of socioeconomic interests^28,31^. In any case, collection of suitable longitudinal data to support model development will need to be a high priority for any future applications of the approach.

## Conclusion

Social influence models have been used extensively as a theoretical tool to better understand processes contributing to the development of social conflict. Our study has now demonstrated their utility as a strategic tool able to accurately hindcast the development of conflict, including catastrophic tipping points, and evaluate strategies aimed at reducing conflict levels. Results indicate that while persuasive advocacy may shift average opinion, it is also likely to increase overall conflict levels. Whereas meaningful engagement, collaborative learning and improving scientific literacy targeted broadly across the population can be effective in both moderating opinions and reducing conflict. While the underlying methods and lessons on effective communications have been demonstrated in the context of historical development of salmon aquaculture, they are likely to be relevant to a broad range of environmental conflicts.

## Supplementary Information

Supplementary Information.

## Data Availability

The model forcing and output datasets generated during the current study are provided as Supplementary material.
