# Supplementary material for: Reducing socio-ecological conflict using social influence modelling
Source: Sci Rep. 2022 Dec 20;12:22002. doi: 10.1038/s41598-022-26570-8 (PMC9768146; doi:10.1038/s41598-022-26570-8)
Supplement: Supplementary file 1 — Supplementary Information. [file 41598_2022_26570_MOESM1_ESM.docx]

**Extended Data**

Model equations

The Social Influence and Event Model (SIEM) builds on the seminal Hegselmann-Krause (HK) bounded confidence model^35^ and has been described previously^6^. Key variables and equations are listed in Table S1 and Table S2 respectively.

**Table S1: Definitions and values of SIEM variables.**

| Variable | Definition | Estimation | Range |
| --- | --- | --- | --- |
| *Individual characteristics* | | | |
| $\boldsymbol{O}_{\boldsymbol{i,t}}$ | Opinion of individual $i$ at time $t$ | Equation S1 | $\left[ -1 1 \right]$ |
| $\boldsymbol{C}_{\boldsymbol{i,t}}$ | Certainty of individual $i$ in their opinion at time $t$ | Stochastic variation around distributions (Table S3, Figure S1) | $\left[ 0 1 \right]$ |
| $\boldsymbol{J}_{\boldsymbol{t}}$ | Set of individuals with which $i$ interacts (including $i$ itself) at time $t$ | Randomly selected with average of $\kappa$ members | $\left\vert I_{t} \right\vert\in\left\{ 0.. 2k+1 \right\}$ |
| $\boldsymbol{w}_{\boldsymbol{ij,t}}$ | Weighting influence of individual $j$ on individual $i$ at time $t$ | Equation S4 | $\left[ 0 1 \right]$ |
| *Event characteristics (media or interventions)* | | | |
| $\boldsymbol{E}_{\boldsymbol{t}}$ | Strength of an event occurring at timestep $t$ | Newspaper data | $\left[ 0 1 \right]$ |
| $\boldsymbol{v}_{\boldsymbol{ik,t}}$ | Weighting influence of event $k$ on individual $i$ at time $t$ | Equation S5 | $\left[ 0 1 \right]$ |
| *System characteristics* | | | |
| $\boldsymbol{P}$ | Total number of individuals in population | Sensitivity runs (Table S3, Figure S2) | $175, 350, 700$ |
| $\boldsymbol{\kappa}$ | Number of connections per individual per timestep or network degree | Sensitivity runs (Table S3, Figure S3) | $\left[ 0 10 \right]$ |
| $\boldsymbol{\varepsilon}$ | Confidence threshold (defining level of homophily) | Sensitivity runs (Table S3, Figure S4) | $0.3, 0.4, 0.5$ |
| $\boldsymbol{\Delta O}_{\boldsymbol{t}}$ | Conflict level at timestep $t$ | Standard deviation of $O_{i,t}$ | $\left[ 0 1 \right]$ |

**Table S2: SIEM equations.**

| Equation | Definition | Number |
| --- | --- | --- |
| $\boldsymbol{O}_{\boldsymbol{i,t+1}}\boldsymbol{=}\sum_{\boldsymbol{j\in}\boldsymbol{J}_{\boldsymbol{t}}} {\boldsymbol{w}_{\boldsymbol{ij,t}}\boldsymbol{O}}_{\boldsymbol{j,t}}\boldsymbol{+}\sum_{\boldsymbol{k\in}\boldsymbol{K}_{\boldsymbol{t}}} {\boldsymbol{v}_{\boldsymbol{ik,t}}\tilde{\boldsymbol{O}}}_{\boldsymbol{k,t}}$ | Opinion equation | S1 |
| $\boldsymbol{J}_{\boldsymbol{t}}\boldsymbol{=}\left\{ \boldsymbol{j \vert}\left\vert\boldsymbol{O}_{\boldsymbol{i,t}}\boldsymbol{-}\boldsymbol{O}_{\boldsymbol{j,t}} \right\vert\boldsymbol{<\varepsilon} \right\}$ | Bounded confidence assumption for individuals | S2 |
| $\boldsymbol{K}_{\boldsymbol{t}}\boldsymbol{=}\left\{ \boldsymbol{k \vert}\left\vert\boldsymbol{O}_{\boldsymbol{i,t}}\boldsymbol{-}{\tilde{\boldsymbol{O}}}_{\boldsymbol{k,t}} \right\vert\boldsymbol{<\varepsilon} \right\}$ | Bounded confidence assumption for events | S3 |
| $\boldsymbol{w}_{\boldsymbol{ij}\boldsymbol{,}\boldsymbol{t}}\boldsymbol{=}\left\{ \begin{aligned} \frac{\boldsymbol{1}}{\left\vert\boldsymbol{J}_{\boldsymbol{t}} \right\vert} \mathbf{if} \boldsymbol{C}_{\boldsymbol{i}\boldsymbol{,}\boldsymbol{t}}\boldsymbol{\leq}\frac{\boldsymbol{1}}{\left\vert\boldsymbol{J}_{\boldsymbol{t}} \right\vert}\sum_{\boldsymbol{j}\boldsymbol{\in}\boldsymbol{J}_{\boldsymbol{t}}} \boldsymbol{C}_{\boldsymbol{j}\boldsymbol{,}\boldsymbol{t}} \\ \boldsymbol{0}\mathbf{if}\boldsymbol{C}_{\boldsymbol{i}\boldsymbol{,}\boldsymbol{t}}\boldsymbol{>}\frac{\boldsymbol{1}}{\left\vert\boldsymbol{J}_{\boldsymbol{t}} \right\vert}\sum_{\boldsymbol{j}\boldsymbol{\in}\boldsymbol{J}_{\boldsymbol{t}}} \boldsymbol{C}_{\boldsymbol{j}\boldsymbol{,}\boldsymbol{t}} \end{aligned} \right.$ | Weighting function for influence of individual $\left( j \right)$ on individual $\left( i \right)$ | S4 |
| $\boldsymbol{v}_{\boldsymbol{ik,t}}\boldsymbol{=}\left\{ \begin{aligned} \boldsymbol{E}_{\boldsymbol{t}} \mathbf{if} \boldsymbol{C}_{\boldsymbol{i,t}}\boldsymbol{\leq}{\tilde{\boldsymbol{C}}}_{\boldsymbol{k,t}} \\ \boldsymbol{0}\mathbf{otherwise} \end{aligned} \right.$ | Weighting function for influence of a media event or intervention event $\left( k \right)$ on individual $\left( i \right)$ | S5 |

Model sensitivity

Additional ensemble model runs were used to identify the sensitivity of results to the distribution of certainty across the population (Table S3, Figure S1); to the size of the total population (Table S3, Figure S2); to the connectivity of the population (Table S3, Figure S3); and to homophily within the population (Table S3, Figure S2).

**Table S3: Sensitivity of Opinion and model results to: distributions of certainty across the population; population size; population connectivity expressed as average number of connections per individual per month (network degree); and homophily within the population expressed as the confidence threshold. Comparisons of model results with surveys for opinion and conflict are represented by the normalised root-mean-square error (NRMSE) where normalisation was with respective to their range (2 for opinion and 1 for conflict); Spearman’s correlation coefficient with p-values; and Pearson’s correlation coefficient with p-values.**

| **Description** | **Certainty distribution**  ^Pop^  ^Certainty^ | **Population size** | **Average network degree** | **Confidence threshold** | **Opinion** | | | **Conflict** | | |
| --- | --- | --- | --- | --- | --- | --- | --- | --- | --- | --- |
|  |  |  |  |  | NRMS  error | Spearman  *r*  (*p* value) | Pearson  *r*  (*p* value) | NRMS  error | Spearman  *r*  (*p* value) | Pearson  *r*  (*p* value) |
| Certainty uniform |  | 350 | 5 | 0.4 | 0.177 | 0.6  (0.350) | 0.644  (0.241) | 0.153 | 0.7  (0.233) | 0.786  (0.115) |
| Certainty split |  | 350 | 5 | 0.4 | 0.153 | 0.6  (0.35) | 0.62  (0.196) | 0.089 | 0.7  (0.233) | 0.881  (0.0486) |
| Certainty low |  | 350 | 5 | 0.4 | 0.133 | 0.7  (0.233) | 0.707  (0.182) | 0.073 | 1.0  (0.0167) | 0.975  (0.0047) |
| Certainty central |  | 350 | 5 | 0.4 | 0.168 | 0.7  (0.233) | 0.619  (0.266) | 0.094 | 1.0  (0.0167) | 0.927  (0.0235) |
| Population halved |  | 175 | 5 | 0.4 | 0.146 | 0.7  (0.233) | 0.741  (0.152) | 0.047 | 0.9  (0.0833) | 0.941  (0.0169) |
| Population doubled |  | 700 | 5 | 0.4 | 0.171 | 0.6  (0.350) | 0.574  (0.312) | 0.086 | 0.6  (0.350) | 0.742  (0.151) |
| Connectivity reduced |  | 350 | 2 | 0.4 | 0.108 | 0.7  (0.233) | 0.772  (0.127) | 0.139 | 1.0  (0.0167) | 0.905  (0.0345) |
| Connectivity increased |  | 350 | 10 | 0.4 | 0.148 | 0.7  (0.233) | 0.736  (0.156) | 0.0623 | 0.9  (0.0833) | 0.935  (0.0196) |
| Homophily decreased |  | 350 | 5 | 0.3 | 0.196 | 0.9  (0.083) | 0.756  (0.139) | 0.086 | 0.8  (0.133) | 0.838  (0.076) |
| Homophily increased |  | 350 | 5 | 0.5 | 0.356 | -0.1  (0.95) | -0.277  (0.652) | 0.334 | 0.3  (0.683) | 0.512  (0.378) |

| **a.** | 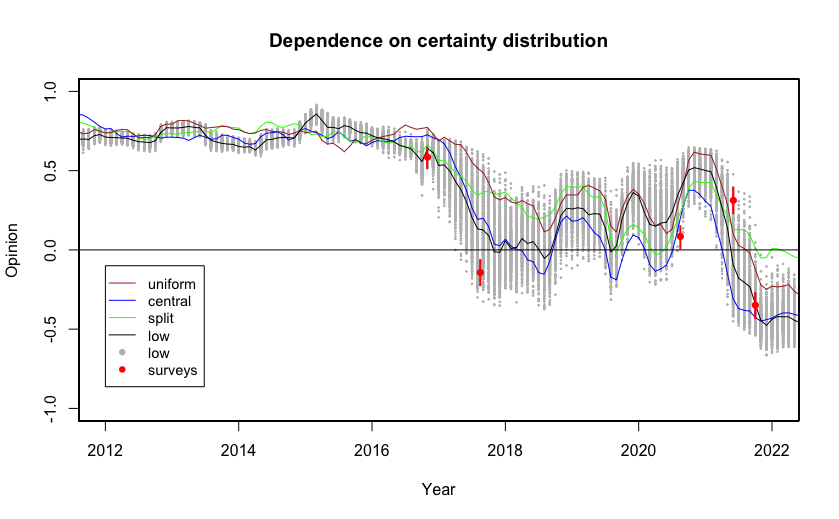 |
| --- | --- |
| **b.** | 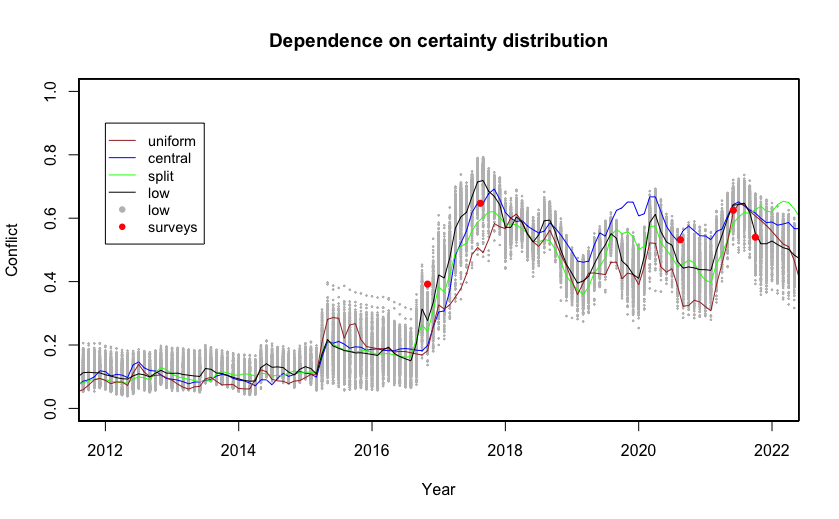 |
| **Figure S1:** (a) Modelled opinion and (b) modelled certainty for alternative distributions of certainty across the population (Table S3). Average opinion (with 95% confidence intervals) and average conflict estimated from five community surveys are also shown for comparison (red points). | |

| **a.** | 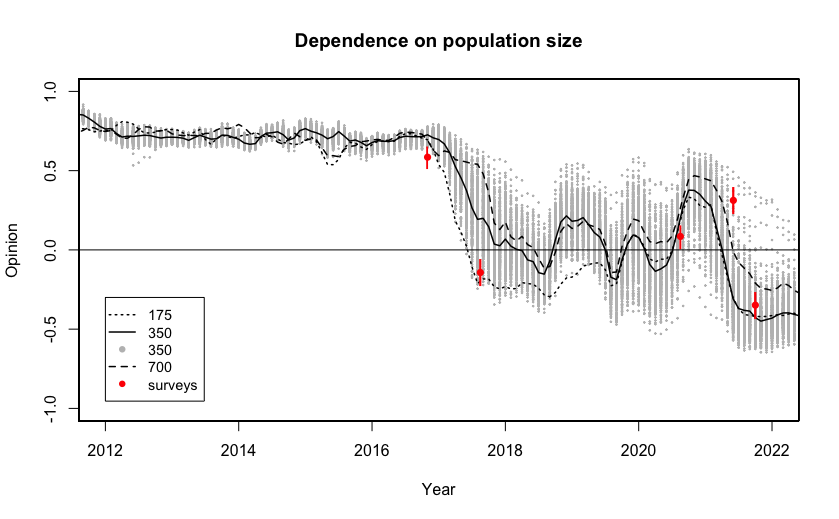 |
| --- | --- |
| **b.** | 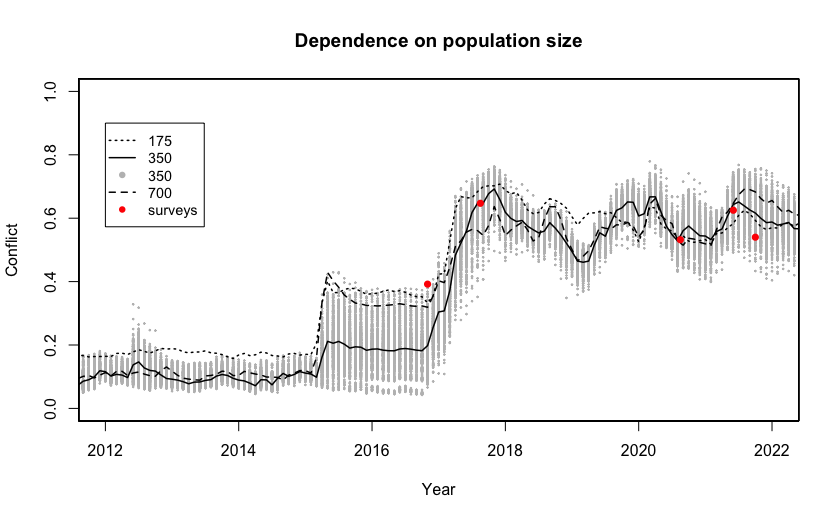 |
| **Figure S2:** (a) Modelled opinion and (b) modelled certainty for a range of population sizes (Table S1). Average opinion (with 95% confidence intervals) and average conflict estimated from five community surveys are also shown for comparison (red points). | |

| **a.** | 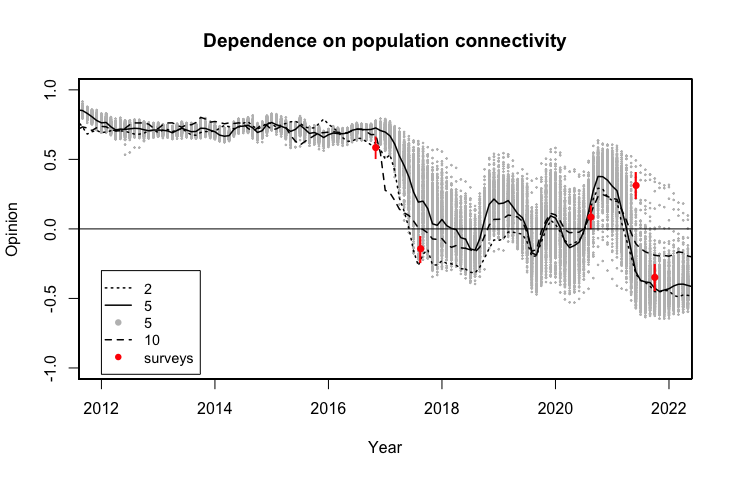 |
| --- | --- |
| **b.** | 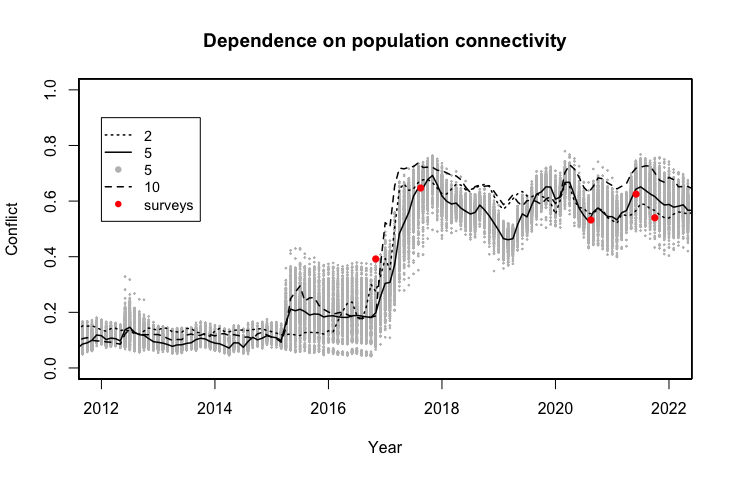 |
| **Figure S3:** (a) Modelled opinion and (b) modelled certainty for a range of connectivity levels expressed as the average number of connections per individual per month or network degree (Table S1). Average opinion (with 95% confidence intervals) and average conflict estimated from five community surveys are also shown for comparison (red points). | |
| **a.** | 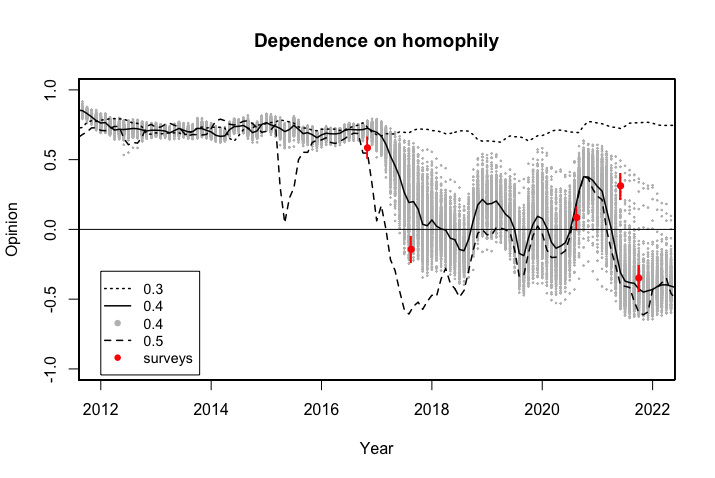 |
| **b.** | 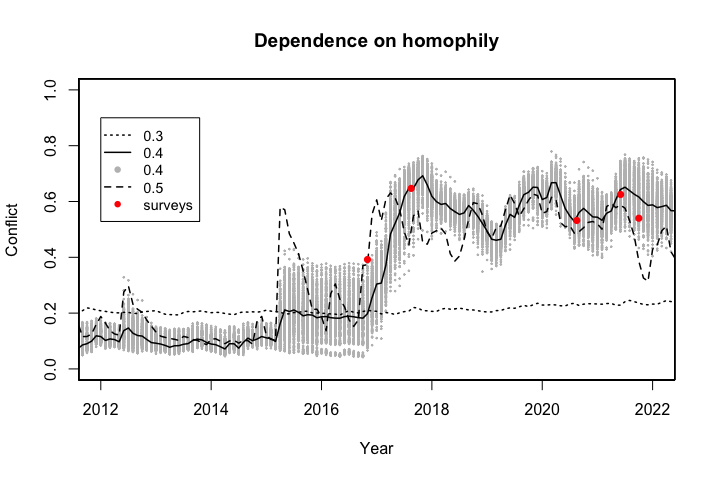 |
| **Figure S4:** (a) Modelled opinion and (b) modelled certainty for a range of homophily levels expressed as confidence threshold values (Table S1). Average opinion (with 95% confidence intervals) and average conflict estimated from five community surveys are also shown for comparison (red points). | |

Evaluating communication strategy performance in reducing conflict

Time series of modelled opinion and modelled conflict are shown in Figure S5 for the strategy with the largest change in mean opinion (positive persuasion) and the strategy with the largest reduction in conflict (meaningful engagement and collaborative learning). The latter was moderately effective in moving opinions towards neutral, whereas positive persuasion tended to increase conflict. Temporally averaged responses to all strategies are presented in Figure 3.

| **a.** | 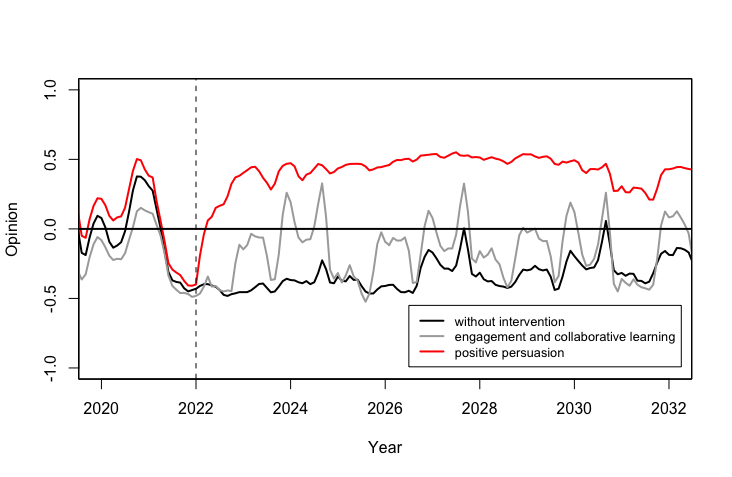 |
| --- | --- |
| **b.** | 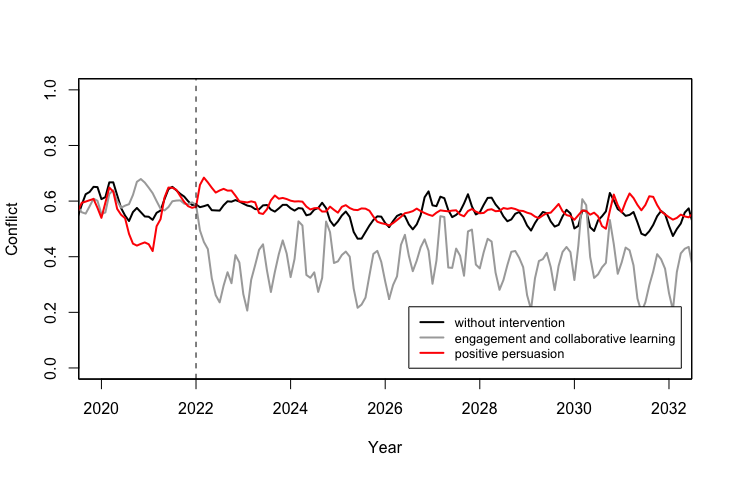 |
| **Figure S5:** (a) Modelled opinion and (b) modelled certainty for a strategy of positive persuasion (red lines) and a strategy of meaningful engagement and collaborative learning (grey lines). Both are compared with not intervening (black line). | |
